# Linking two randomised controlled trials for Healthy Beginnings©: optimising early obesity prevention programs for children under 3 years

**DOI:** 10.1186/s12889-019-7058-9

**Published:** 2019-06-13

**Authors:** Li Ming Wen, Chris Rissel, Huilan Xu, Sarah Taki, Wendy Smith, Karen Bedford, Alison J. Hayes, Philayrath Phongsavan, Judy M. Simpson, Miranda J. Shaw, Renee Moreton, Louise A. Baur

**Affiliations:** 1 Health Promotion Unit, Population Health, Sydney Local Health District, Level 9, King George V Building, Missenden Road, Camperdown, Sydney, New South Wales 2050 Australia; 2 Sydney School of Public Health, Faculty of Medicine and Health, and the Charles Perkins Centre, the University of Sydney, Camperdown, Sydney, New South Wales Australia; 3 NHMRC Centre of Research Excellence in the Early Prevention of Obesity in Childhood (EPOCH), Charles Perkins Centre, The University of Sydney, Camperdown, Sydney, New South Wales Australia; 4 NSW Office of Preventive Health, Ministry of Health, St Leonards, Sydney, New South Wales Australia; 5 Discipline of Child & Adolescent Health, The University of Sydney, Camperdown, Sydney, New South Wales Australia; 6 Community Health Services, Sydney Local Health District, Camperdown, Sydney, New South Wales Australia; 7 Sydney Institute for Women, Children & their Families, Sydney Local Health District, Camperdown, Sydney, New South Wales Australia

**Keywords:** Randomised controlled trial, Obesity, Prevention, Children, Telephone consultation, Text messaging, Body mass index

## Abstract

**Background:**

Beginning in 2017 we have conducted a 3-arm randomised controlled trial (RCT) to determine the effectiveness of an early obesity intervention in the first two years of life using either telephone or Short Message Service (SMS) support for mothers. The trial recruited 1155 mothers from their third trimester of pregnancy. This protocol is for a new trial to build on the existing trial using the mother-child dyads retained at 24 months for recruitment to the new RCT. The aim of this new trial is to test whether use of a combination of telephone and SMS interventions is effective in promoting healthy eating and physical activity, as well as reducing child body mass index (BMI) at 3 years of age.

**Methods:**

We will conduct a parallel RCT with an estimated sample of 750 mother-child dyads retained from the existing trial at 24 months. Mothers who completed the 24 months survey, including a telephone survey and measurement of child’s height and weight will be invited to participate in the new trial. Informed consent will be obtained at the 24 months survey. The participating mother-child dyads will then be randomly allocated to the intervention (combined telephone and text messaging intervention) or the control group. The intervention will comprise three staged telephone consultations and text messages after each of the three intervention booklets is mailed to mothers at specific time-points between two and three years of child age. The main trial outcome measures include a) BMI and BMI z-score measured at 36 months, b) diet, physical activity and screen time c) cost-effectiveness, and d) feasibility and acceptability of the intervention.

**Discussion:**

This unique opportunity to link two studies will expedite project start up time, utilise existing research infrastructure and systems to run the study, and optimise the use of an already engaged population of study participants. It can address a significant knowledge gap regarding early obesity prevention for children aged 2 to 3 years. The feasibility and effectiveness of the combined telephone and SMS intervention will indicate whether this is a scaleable, broad-reach and low-cost early obesity intervention.

**Trial registration:**

The trial was registered with the Australian Clinical Trial Registry (ACTRN12618001571268) on 20/09/2018.

**Electronic supplementary material:**

The online version of this article (10.1186/s12889-019-7058-9) contains supplementary material, which is available to authorized users.

## Background

Ending Childhood Obesity is now a global health policy issue for the World Health Organisation [1]. Worldwide, the prevalence of childhood obesity increased around eight-fold in both girls (0.7 to 5.6%) and boys (0.9 to 7.8%) between 1975 and 2016 [2], with 41 million children under the age of 5 affected by overweight or obesity in 2016 [1]. About one in five Australian children aged 2–3 years [3] and one third of children aged 2–5 years [4] are affected by overweight or obesity. Promoting healthy feeding and physical activity, together with reducing screen time, are fundamental to preventing obesity among children in the first few years of life and consistent with WHO guidelines on childhood obesity prevention [1].

Although it is increasingly argued that childhood obesity prevention should begin in the early years, current evidence of effective, cost-effective and sustainable interventions is scarce. Not surprisingly, no quality evidence is found specifically targeting 2–3 year old children. Of the few intervention studies we found for children less than 5 years, most targeted children either from birth to 2 years (with an increasing focus on the first 1000 days of life) [5–7] or from 3 to 5 years of age using childcare-based interventions [8]. The most frequent intervention duration was 6 months, with outcomes focussed on dietary and physical activity behaviours rather than weight status. Very few studies used short message service (SMS) or telephone support as the main form of early intervention [1, 7]. In Australia most state health departments focus their early obesity prevention program through formal childcare services. For example, the ‘Munch & Move’ program in the state of New South Wales (NSW) aims to promote and encourage healthy eating and physical activity habits and reduce small screen recreation in young children from birth to 5 years who attend early childhood education and care services [9]. However, the Australian Bureau of Statistics showed that about 48% of 2-year-old children and 44% of 3-year-old children did not attend formal childcare services in 2014 [10]. Therefore, interventions that focus solely on childcare services may miss a large proportion of children in the target age group.

The Healthy Beginnings© program is an early obesity prevention program delivered by Child and Family Health Nurses originally through home visits in the first two years of life [11–15]. Healthy Beginnings© was found to be effective in reducing child body mass index (BMI) at 2 years of age [13], but may not be sustained in the longer term [15] nor cost-effective [16]. There is emerging evidence that interventions delivered through telephone-based counselling [17, 18] or SMS improve health behaviour [19, 20]. Since 2017, we have conducted a 3-arm randomised controlled trial (RCT) to evaluate the efficacy of using SMS or telephone support, plus mailed educational materials for mothers with newborns, as an alternative delivery model for the Healthy Beginnings© early intervention program (http://www.healthybeginnings.net.au/). The study protocol titled “A 3-arm randomised controlled trial of Communicating Healthy Beginnings Advice by Telephone (CHAT) to mothers with infants to prevent childhood obesity” was published in early 2017 [21]. Known as the CHAT trial, the interventions are being delivered from the third trimester to 2 years of age. The interventions include nine staged mail-outs over 24 months addressing key developmental milestones, followed by either nine staged telephone-support sessions by a Child and Family Health Nurse (six between the 3rd trimester to 12 months and three from 12 to 24 months), or nine sets of SMS messages. Each SMS set comprises eight messages which are sent out twice a week over a 4-week period. However, as stated in the published study protocol on the study design, further funding would be required to assess the outcomes at 24 months.

Based on the preliminary findings from the CHAT trial several advantages of using telephone support as a stand-alone program have been identified. Telephone support was seen by the mothers as more personal and increased the mother’s self-reported likelihood of identifying and addressing challenges with practising the desired health behaviours that influence healthy weight gain (detailed findings to be reported elsewhere). However, these advantages can be reduced by the time and effort spent contacting the participants. On average, the intervention nurse has needed to make 6–10 call attempts to each participant for one successful telephone support session, which is not an efficient use of resources (nurse time). On the other hand, using SMS has advantages as an efficient, simple and lower-cost method to deliver information to mothers and to respond quickly to their queries. Participants can also review responses at a time of their convenience. This is particularly useful for mothers with strong demands on their time. Despite the fact that the text messages were bi-directional, there was limited capacity for the intervention nurses to gather a detailed understanding of the challenges being experienced by mothers through SMS, and responses to mothers were likely to be brief. Thus, the combined intervention (Tel + SMS) being investigated in this new study seeks to mirror a real-life situation where interventions such as telephone support rarely occur in isolation and are often accompanied by SMS reminders and missed call messages. By combining telephone and SMS into an intervention delivery, we will be able to compensate for inherent limitations of each element and greatly improve their collective effectiveness.

**Fig. 1:**
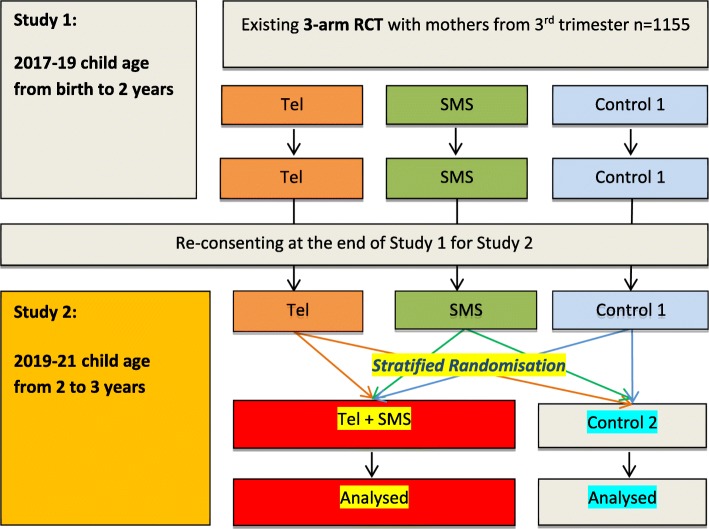
Study Designs of Study 1 and 2

Therefore, in this study protocol we are undertaking a new RCT which will be linked with the existing 3-arm RCT using the same study participants (see Fig. 1). This approach will enable us to use the 24-month survey for dual purposes in the existing 3-arm CHAT RCT (Study 1) and the new RCT (Study 2). For Study 1, the 24-month survey will measure the intervention outcome and effectiveness of either the telephone support intervention or the SMS intervention. For Study 2, the 24-month survey will be an integral part of the recruitment process and baseline data collection for this new RCT in determining the effectiveness of a combined telephone and SMS intervention.

### Aims and hypotheses

The aim of the study is to evaluate the effectiveness and cost-effectiveness of combined telephone and SMS intervention targeting mothers of children between 2 and 3 years of age in promoting healthy eating and physical activity, as well as reducing child BMI at 3 years of age.

We hypothesise that, in comparison with usual care, the Tel + SMS intervention will improve the following main outcomes:

- lower child BMI at age 3 years;
- lower child screen time (i.e. TV viewing time) and increased physical activity;
- better dietary quality (i.e. greater intake of fruits/vegetables) at 3 years;
- improved mothers’ healthy eating and physical activity; and
- demonstrated cost-effectiveness of the intervention.

The study aims to specifically address the following research questions:

1. *Will a combined telephone and SMS support early intervention with mailed health resources delivered to mothers with children between 2 to 3 years significantly reduce children’s BMI at age 3 years?*
2. *Will a combined telephone and SMS support early intervention with mailed health resources delivered to mothers with children between 2 to 3 years significantly improve child eating behaviours, and significantly decrease the prevalence of obesity-related risk behaviours at 3 years of age?*
3. *What are the incremental costs and benefits of the combined telephone and SMS support early intervention from a health funder perspective?*
4. *Is the intervention feasible and acceptable and to what extent is the intervention likely to be translated into practice?*

## Methods

### Design

A parallel RCT using the study participants from the existing CHAT trial (Fig. 1).

### Setting

The study will be carried out in four local health districts in New South Wales, Australia, including the Sydney, South Eastern Sydney, South Western Sydney and Southern NSW Local Health Districts.

### Participants and recruitment

**Fig. 2:**
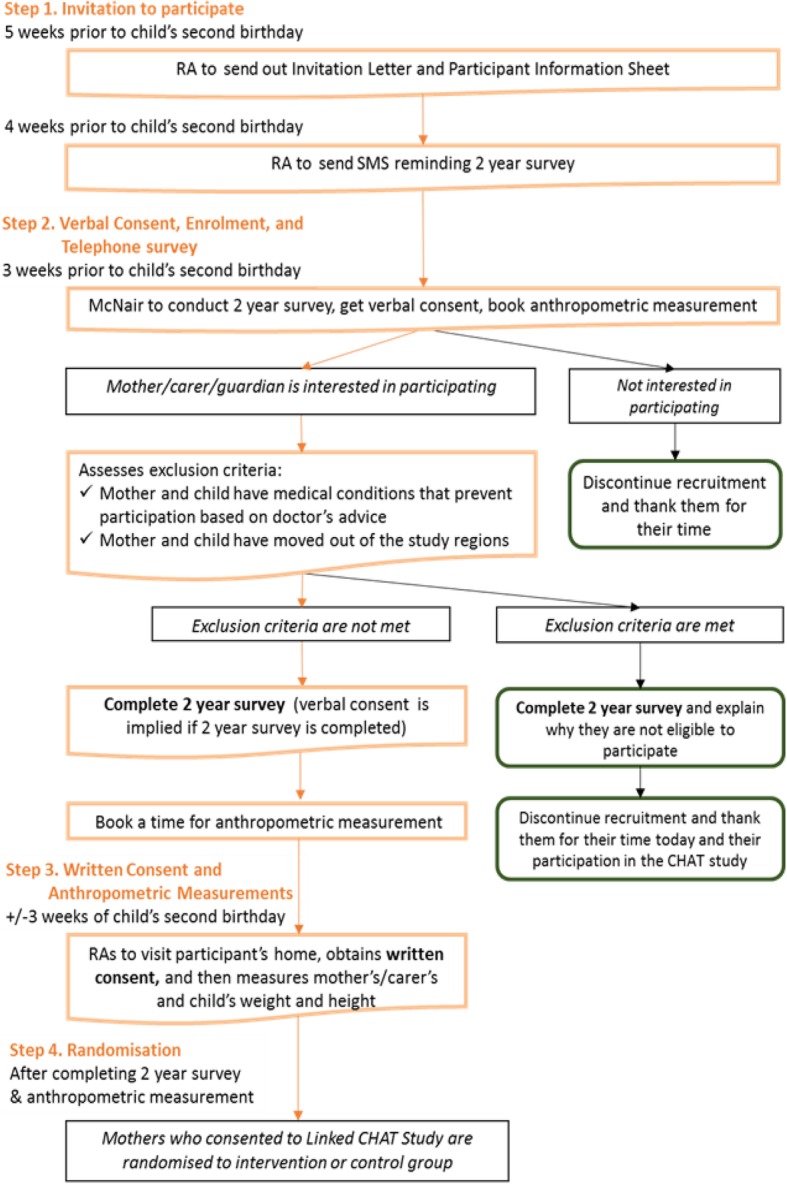
Recruitment flow chart

As shown in Fig. 2, 5 weeks before the participating child turns 2 years old in the existing CHAT trial, their mother or primary caregiver will be sent a letter of invitation with information about the 24-month survey and Study 2. A week later, a text message will be sent out to remind participants of the 24-month survey. Three weeks before the child reaches 2 years, a market survey interviewer will contact participants on behalf of the project team to conduct the 24-month telephone survey. A telephone script will be used to ensure consistency and documentation across all interviewers. Participants will be asked to verbally consent to participation prior to commencing the survey. Once verbal consent is obtained, the interviewer will conduct the 24-month survey (i.e. baseline for Study 2) using the computer-assisted telephone interview (CATI).

During the last section of the telephone survey, interviewers will invite participants to receive a home visit by a research assistant (RA) for face-to-face measurement of their own and their child’s height and weight. Verbal consent will be initially requested by the interviewer, however, once the RA has organised the home visit, they will ask for the participant’s written consent before conducting the measurements. Overall, participants will be offered the following options for participation:

1. Only consent to complete the 24-month telephone survey
2. Consent to participate in the 24-month telephone survey and the home visit for the anthropometric measurements
3. Consent to participate in options 1 and 2, and also the new RCT to receive the intervention at 2 to 3 years of age; or
4. No consent to participate in any component.

When the RA and the participating mother meet face-to-face to measure her and her child’s height and weight, the RA will first revisit the Participant Information Sheet and Consent Form and answer any questions that the participant may have. The RA will then obtain the participant’s written consent for the new trial to support the verbal consent already obtained.

### Inclusion criteria

Inclusion for Study 2 is limited to participants of the existing CHAT trial, in which the participating women at the time of original recruitment were aged 18 years and over, were at 28–34 weeks of pregnancy, were able to communicate in English, had a mobile phone, lived in the recruitment areas, were able to give informed consent and did not have any severe medical conditions based on advice given by their doctors.

### Exclusion criteria

Mothers and children will be excluded from the study if they have moved out of the study area, or they have developed a severe medical condition since commencing the study.

### Sample size

For the existing Study 1 the sample size required was previously presented in the published research protocol (1056 mothers: 352 in each of the 3 arms of the study) [21]. A total of 1155 mothers were recruited into the trial exceeding the required sample size by 100 mothers. We originally estimated that a total sample size of 792 (264 per arm × 3) was needed at 2 years to have sufficient power to detect a difference in child BMI of 0.38 kg/m^2^.

For Study 2, we will need a total sample size of 506 (253 per arm × 2) by the end of the study (at 3 years of age). This would allow us to detect a difference of 0.40 kg/m^2^ in mean BMI (SD = 1.60 based on Healthy Beginnings Trial) at the 2-sided 5% significance level with 80% power. This effect size is achievable based on the findings from a 6-month intervention study in the US that detected a decrease in BMI of 0.40 kg/m^2^ with children aged 2–5 years [22]. Allowing for a conservative 15% loss to follow-up between 2 and 3 years, we will need around 600 participants at 2 years of age. This sample size will also give enough power to detect differences in other main outcomes (e.g., a difference of 0.3 in the mean number of daily serves of fruit or vegetables).

### Randomisation

After completing the 24-month (baseline) survey and measurement, participating mothers will be randomly allocated to either the intervention (Tel + SMS) or the control group (Control 2). Randomisation will take place using a web-based tool (http://www.randomization.com/). The intervention allocation scheme will be generated using random permuted blocks stratified by the group allocation in Study 1 (see Fig. 1). The use of stratified randomisation is to ensure that any carry-over effect of the CHAT intervention of Study 1 will be equally distributed between treatment groups in Study 2.

### Intervention (Tel + SMS) protocol

The intervention will be guided by the Health Belief Model [23], and uses motivational interviewing techniques to facilitate behaviour change [18]. The intervention aims to improve mothers’ parenting behaviours and set up healthy behaviours (i.e., healthy feeding practices, active play and reduced screen time). Telephone support will consist of protocol-based sessions. A telephone protocol and manual will be developed to ensure consistency of intervention delivery based on our current trial. The main content is in keeping with the Australian Dietary Guidelines, early childhood developmental guidelines and the Australian 24-Hour Movement Guidelines for the Early Years (Birth to 5 years) [24]. Telephone sessions will be delivered by a clinician (Child and Family Health Nurse/midwife) trained in telephone support, health behaviour change strategies, and child development. Based on our current trial experience, we anticipate that each telephone session will be ~ 20 min in duration.

The Tel + SMS intervention will be delivered between age 2 and 3 years (see Table 1). Once random allocation is completed, the intervention group will receive scheduled support across 3 time-points (24–28, 28–32, and 32–36 months). At each time-point, participants will be sent one of the three intervention booklets that provide information on key developmental milestones associated with promoting behaviours for healthy weight gain. The intervention booklets will be developed according to the key messages (see Table 1) that support child development during this age of a child’s life. Following a mailed intervention booklet, participants will receive a telephone support call from a research clinician. The clinicians will employ a motivational interviewing approach and aim to set goals, build skills and knowledge, address barriers, and manage any anxiety or stress which may act as a barrier to health behaviour change.

**Table 1:** Main focus and contents of the staged early intervention and sample of the text messages

Component/focus	Main contents	Sample of SMS
Repeated exposure to healthy foods, limiting exposure to non-core foods	Recommended dietary intake of food groups, food label reading, healthy food environments, eating family meals, oral health, managing external influences on dietary intake	“Messy eating and playing with food are normal parts of [baby’s name] development when learning to eat independently. Check out the Healthy Beginnings booklet for tips to help with feeding”
Promotion of choosing water as a drink and exclude sugar sweetened beverages	Encourage drinking healthy drinks from a cup, oral health	“Drinking water from a cup is the only drink that [baby’s name] needs. Fruit juice, soft drinks, flavoured milk can cause teeth decay”
Promotion of responsive complementary feeding strategies	Hunger and satiety cues, strategies to manage fussy eating behaviours	“At 2–3 years it is normal for toddlers’ appetites to go up and down. Keep on providing a variety of healthy foods and it is up to [baby’s name] to decide how much food to eat.”
Promotion of incorporating physical activity into children’s daily routine	Encourage active play, support the development of Fundamental Movement Skills, developmental milestones, child safety	“It is recommended that toddlers should be active every day for at least 3 h throughout the day. Playing in the park, kicking a ball or catching & throwing are some fun activities”
Promotion of reduction in any screen-time (TV, DVDs, computers, smartphones, iPads/tablets)	Avoid any screen time, encourage quiet (reading, singing, music, drawing, dress-ups) and active (dancing, playground, running, walking)	“Your baby needs to be active every day and does not need any screen time. Playing with other children in a safe, supervised area will help them develop socially.”
Promotion of fostering healthy sleeping habits	Developing a sleep schedule, developing a bedtime routine, understanding sleep patterns	“At 2–3 years toddlers need to sleep between 12 and 13 h a day. The Healthy Beginnings booklets have tips on how to develop a healthy sleep routine. For more support send us a text”

Key Features of the intervention delivery mode:

• The intervention will be delivered at 24–26, 28–30, and 32–34 months

• A Healthy Beginnings booklet will be sent to participants which provides information on relevant topics for each milestone

• Two SMSs will be sent per week for 8 weeks tailored to the child’s development and main focus

• Three telephone calls using Anticipatory Guidance to deliver the content at each intervention stage by trained Child and Family Health Nurses

In addition, participants will also receive two text messages per week for the next 8 weeks (i.e., 16 SMS messages to enhance and reinforce the support provided in the intervention booklet and telephone session). The text messages will be generated and sent using a dedicated, secured web-based software that was used in the existing trial (Study 1). This software has the ability to automate, schedule and personalise the text messages delivered (based on the child’s age) to deliver age appropriate information and to address the message using the child’s and mother’s name. Information can be further tailored at any point during the intervention. Further, a key feature of the SMS program is that the messages are bi-directional so mothers are able to respond to text messages sent and receive a response from the clinicians delivering the program. In addition to sending SMSs on key developmental milestones, mothers will also be reminded of booking telephone calls with our research clinicians. Incoming SMSs will be reviewed daily (except for weekends and public holidays) by the research clinicians.

### Control arm

In the control group mothers will receive usual care, which all mothers (including the intervention mothers) receive if needed from the service. Further, this group will also receive two booklets that do not contain any information relating to the intervention resources. The booklets will provide information on other aspects of child development such as toilet training, language development and sibling relationships. This strategy is used to maximise mothers’ engagement in the study and to reduce attrition rates.

### Retention strategies

We have developed a set of effective cohort retention strategies through the existing CHAT trial [21]. For example, we will send a thank-you card and birthday card for all participating family and children. After hours telephone support calls will be available for mothers who have been back to work in the intervention group.

### Data collection

The 24-month survey will be collected by computer-assisted telephone interviewing (CATI) and home visiting for anthropometric measurements by an RA who is blinded to treatment allocation.

### Primary outcomes

The primary outcome is child’s BMI (weight/length^2^; kg/m^2^) or BMI z-score at 3 years of age as used in the previous Healthy Beginnings Trial [13, 15]. Child’s height and weight (and hence BMI, or BMI z-score) will be measured according to the measurement protocol (Additional file 1) in the home by RAs blinded to treatment allocation. Two measures of height will be taken to the nearest 0.1 cm using a portable stadiometer. Measurements of weight (to the nearest 0.1 kg) will be taken using digital scales with the child wearing light clothing and no shoes.

### Secondary outcomes

At 3 years, secondary outcomes will include child’s and mother’s screen-based activities (e.g., watching TV), fruit and vegetable consumption and other eating habits.

Child’s eating habits and screen-based activities: These measures will be reported by their mother using a validated short food frequency questionnaire (FFQ) and validated activity questions [25, 26].

Mother’s nutrition and physical activity: These outcomes will be assessed based on the questions from the NSW Health Survey Program questionnaire which was used previously in the Healthy Beginnings Trial [13, 15].

### Socio-demographic characteristics at baseline (24 months)

We will collect or update socio-demographic data if there is any change to demographic characteristics (e.g, employment status, marital status).

### Data analysis

CONSORT guidelines for reporting randomised controlled trials will be followed to analyse and report the results. Intention-to-treat principles will be adopted to analyse all outcomes. The primary analysis for Study 2 will be adjusted for the stratification by Study 1 treatment group, as per CONSORT guidelines. For continuous variables, such as BMI, or BMI z-score, means will be compared using linear regression. For binary categorical variables, logistic regression model will be used. Secondary analyses will additionally adjust for exact age, and also will use mixed model and multiple imputation to assess the effect of loss to follow-up as used in the previous Healthy Beginnings Trial [13]. Mean differences of BMI or BMI z-score between the intervention and control groups and adjusted odds ratio (AOR) with 95% confidence interval (CI) will be reported.

### Issue of ‘contamination’ and ‘carry-over effect’ from study 1 to study 2

To assess the level of contamination in the control group and also to test whether the intervention group is receiving additional information, we will ask mothers in both groups to report their sources of information on healthy child feeding and active play. Study 1 intervention will be included in the multivariable regression models to adjust for its effect. In our previous study we demonstrated that, without further intervention, the early intervention effects diminish over time [15], so any carry-over effect from Study 1 to Study 2 should be small. Even if there is a carry-over effect, it will be balanced between the intervention and control groups by the stratified randomisation.

### Assessor blinding

A CATI market survey company will be used to collect baseline at 2 years or outcome measures at 3 years through telephone interviews. Four RAs who are not involved in intervention implementation will conduct anthropometric measures via home visiting. The RAs and data entry staff will be blinded to treatment allocation. In addition, participating mothers will be blinded to the specific details of the research hypotheses and asked not to disclose treatment allocation.

### Cost-effectiveness analysis (CEA) and cost-utility analysis (CUA)

From a health provider perspective, the cost-effectiveness analysis will be conducted alongside the trial in which primary outcomes will be measured in natural units (e.g. BMI z-score). As supportive interventions for parents are likely to improve parent health-related quality of life, we will also do cost utility analysis. We will examine quality of life of the primary care-giver using the EuroQol 5 dimensions (EQ-5D) [27] at 1 year, 2 years and 3 years, which will allow calculation of quality adjusted life-years (QALYs).

### Analysis of costs

Data will be collected prospectively on the costs of delivering the intervention program (including telephone calls, SMS, the 2-way SMS service, staff time, training, mail outs, and any other intervention resources) from 2 to 3 years. These will only include the costs of all resources needed to reproduce the intervention. The cost of all intervention materials will be based on market prices. The reference year for the analysis will be 2019. All costs will be in Australian dollars and indexed to that year using the Health Price Index and all costs and effects will be appropriately discounted.

### Outcomes for CEA

Similar to a previous evaluation of the Healthy Beginnings Trial [16], BMI and BMI z-score will be used to assess the cost-effectiveness of the intervention compared with the control group at 2 years (for Study 1) and at 3 years (Study 2). Incremental cost-effectiveness ratios will be calculated in terms of the incremental cost: a) per unit BMI reduction per child at 3 years, and 2) per QALY gained. Uncertainty in mean costs, outcomes and ICERs will be determined through bootstrapping. Scatter plots of the joint distribution of incremental costs and effects will be displayed on a cost-effectiveness plane and the probability of the intervention being cost-effectiveness will be determined for different willingness-to-pay thresholds.

### Process evaluation

All aspects of contact (telephone sessions and SMS) with the families will be recorded by the intervention nurses (or clinicians). The project coordinator will provide regular reports to the investigators and project management team about the implementation process and issues arising. All intervention data including recruitment, study retention and intervention acceptability will be collected and stored securely on the REDCap database system [28] hosted by Sydney Local Health District, Australia. Thematic analysis of participants’ responses (de-identified) will be evaluated retrospectively to determine the major themes and issues participants faced during this time in their child’s development. SMS delivery and receipt will be automatically tracked by the program.

### Ethical implications

The two linked trials have already been granted approval by the Ethics Review Committee of Sydney Local Health District (Protocol No. X16–0360 & LNR/16/RPAH/495 and Protocol No X18–0387 & HREC/18/RPAH/545). Both trials are registered with the Australian Clinical Trial Registry (ACTRN12616001470482 and ACTRN12618001571268). Written informed consent will be obtained from all study participants.

### Evaluation of the feasibility and acceptability

We will conduct in-depth interviews over the phone with some participants to assess program feasibility and acceptability including participant retention, satisfaction, and evidence of behaviour change. We will also gather mothers’ feedback on the intervention acceptability, barriers and enablers. All interviews will be audio-recorded and transcribed verbatim. Data will be organised and thematically analysed using the Framework Analysis [29].

### Timeline

Our proposed timeline as shown in Table 2 is 2.5 years including 2 months for study preparation. Our first child (from the existing trial) will be 2 years old in March 2019 and the last child will reach 2 years by the end of September 2019. Thus, baseline data collection will occur over 6 months as children reach their second birthday, with 6 months at the end for data analysis and reporting. All the intervention methods including telephone support and SMS have already been tested in the existing trial and the basic research infrastructure including the research database, telephones and SMS system are all in place and fully functioning.

**Table 2:**
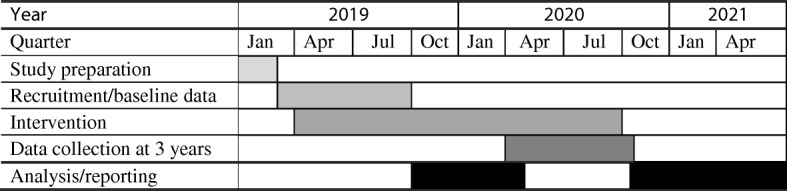
Timeline of the new trial

## Discussion

To our knowledge, the two linked RCTs will be the first to determine the effectiveness and cost-effectiveness of a telephone-delivered, SMS-delivered, or combined (Tel + SMS) program in preventing early onset of childhood obesity. This new trial will greatly add value to the existing CHAT trial and yield two-fold benefit relevant to filling knowledge gaps in early obesity prevention for children aged 0–3 years: 1) enabling the testing of Study 1 hypothesis at 2 years, and 2) a quick access to recruitment from a pool of eligible cooperative study participants for the new RCT (Study 2). It can fill in a significant knowledge gap in early obesity prevention for children aged 2 to 3 years using a widely accepted and easily administered approach with relatively inexpensive methods.

A new combined telephone support and SMS intervention model has the potential to provide an efficacious early obesity intervention approach with broad-reach at relatively low cost. More importantly, this approach can be adapted and adopted according to community needs, in particular to vulnerable and difficult-to-reach families. The two linked trials will generate new knowledge for policy and practice on the appropriateness, effectiveness and cost-effectiveness of the early obesity intervention strategies in Australia and in other international settings. It will provide new knowledge on recruitment, retention and acceptability of early obesity prevention interventions in community settings, and will also provide evidence that can be applied for promoting other positive health behaviours. The results will provide a template for how to scale-up the most cost-effective community-based obesity prevention program that can be disseminated under real world conditions. The study has the potential to enable policy makers to make decisions on which community-based programs to invest scarce public health funds. In addition, while there have been some obesity prevention trials in early childhood, none has used a combination of staged and combined telephone and SMS intervention, a novel aspect of this research.

## Additional file

Additional file 1: Protocol for Anthropometric Measurement. (DOCX 199 kb)

## Data Availability

Not applicable.
