# Supplementary material for: Linking two randomised controlled trials for Healthy Beginnings©: optimising early obesity prevention programs for children under 3 years
Source: BMC Public Health. 2019 Jun 13;19:739. doi: 10.1186/s12889-019-7058-9 (PMC6567558; doi:10.1186/s12889-019-7058-9)
Supplement: Supplementary file 1 — Protocol for Anthropometric Measurement. (DOCX 199 kb) [file 12889_2019_7058_MOESM1_ESM.docx]

**Title: Linking two randomised controlled trials for Healthy Beginnings©: optimising early obesity prevention programs for children under 3 years**

**Protocol for Anthropometric Measurement**

**Purpose** To ensure the reliable, accurate measurement of anthropometry for the Healthy Beginnings Linked CHAT study, at child age 2 and 3 years.

**References** NSW Health Growth Assessment in Children and Weight Status Assessment in Adults – GL2017_021 Guideline, November 2017

Healthy Beginnings Linked CHAT Protocol for Home Visits

Community Health Infection Prevention and Control Policy Compliance CH_SLLHD_PCP2017_116 Procedure <http://slhd-intranet.sswahs.nsw.gov.au/SSWpolicies/pdf/Community%20Health%20SLHD/CH_SLHD_PCP2017_116.pdf>

Community Health SWP

<http://slhdintranet.sswahs.nsw.gov.au/slhd/communityhealth/pdfs/ohs/SWP/SWP045.pdf> Weighing Children in the Home and Outreach

Community Health SWP

<http://slhd-intranet.sswahs.nsw.gov.au/slhd/communityhealth/pdfs/ohs/SWP/SWP107.pdf>

Stadiometer use when measuring height in a clinical setting.

**Resources** Portable digital scale - weigh in 100g (0.1kg) increments

Portable stadiometer

Non- slip mat for under stadiometer

Participant Information Sheet

Written Consent Form

Measurement form

**Equipment**

All research assistants have access to appropriate portable digital scales and stadiometer.

Equipment must be maintained in good working order. The equipment must be serviced (including calibration) according to manufacturers’ guidelines. This is usually annually or more frequently if the scales are moved regularly; or there are concerns about accuracy.

Alcohol based hand rub/gel

Neutral detergent wipes for cleaning of equipment , extra AAA batteries

**Training**

To assure reliable measurements, all research staff first attend a training session for weight and length measurements, instructed by a professional/expert. The training session involves instruction and practical observation, measurement and guidance.

**Procedure**

The research staff will first revisit the Participant Information Sheet and Consent Form and obtain the participant's written consent to support the verbal consent already obtained.

The child's mother/primary caregiver/guardian will be present while the measurements are being taken, and this will be in a private area.

Explain the procedure to the mother/primary caregiver/guardian and the toddler and ask for their assistance. Provide distraction for the toddler if necessary.

**Weight** **measurement for children 2 years and older**

**Preparation**

1. Perform Hand Hygiene (HH)
2. Place scales on a hard, level floor surface (not carpet). Make sure the space is clear.
3. Explain to the child that you are measuring their weight.
4. Make sure that any outer heavy clothing such as a coat, jacket, or jumper is removed. Light clothing can be worn.
5. Remove shoes and socks and ensure pockets are empty.

**Weighing**

1. Turn the scales on and wait until they zero.
2. Ensure scales are set to the kilogram mode
3. Ask the child to stand on the middle of the scales, on the foot print, look straight ahead and stand still.
4. You may need to move them into the right position or ask the parent/carer to do this.
5. Check the child is not holding onto a wall or table; and arms are at their side.
6. Wait until the scales settle at a reading.
7. Repeat steps 1,2,3,4,5. (weigh child twice)
8. Perform HH
9. Record weight on measurement form or in Redcap

**If difficult to measure child alone, measure weight with carer:**

- Ask parent/carer to remove any of their heavy outer clothing and remove shoes and socks.
- Turn the scales on, ensure set to kilograms mode and wait until they zero.
- Ask the parent/carer to stand on the scales and wait until reading settles.
- Record parents/carer’s weight.
- Ask parent/carer to hold child (no heavy clothing, shoes or socks) and stand on scales together, wait until reading settles.
- Record combined weight.

(Child weight= (adult weight) – (combined weight), **Note: research staff doesn’t need to calculate child weight, REDCap database will calculate it automatically**).

**DO twice and record twice**

**Recording**

1. Bend down if necessary using safe work practice to read the scale at eye level
2. Record weight(s) to the nearest 100g (0.1kg)
3. Record measurements and any notes on measurement form and or in Redcap using Ipad

**Height/stature measurement for children 2 years and older**

**Preparation**

1. Place non-slip mat on a hard, flat floor surface (not carpet).
2. Build stadiometer.


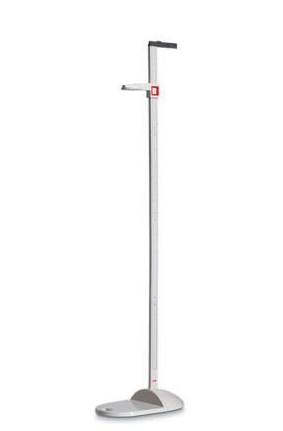

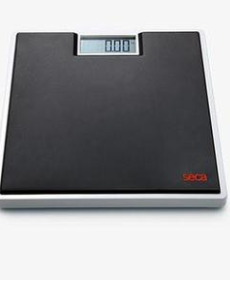


1. Place stadiometer on non-slip mat with measuring pole with lever against a wall.
2. Show the child the stadiometer and explain you are going to see how tall they are. It can be helpful to measure the parent first if the child is hesitant.
3. Take the child over to the stadiometer and make sure they face away from the equipment.
4. Ask Parent/care to remove the child’s shoes and socks.

**Measuring**

1. Perform HH
2. Position the child facing away from the stadiometer (i.e. their back is against the measurement board/stick:
   - with bare feet close together,
   - heels against the heel plate, stand on footprint stickers
   - legs straight,
   - arms at sides and shoulders relaxed.
3. Ask the child to look straight ahead, breathe normally & remain still
4. Double-check their position, as above. Check that corner of eye is level (straight line) with earhole.
5. Bring the measuring device/head plate down to rest on the child’s head.
6. Ask them to step off and on again to measure again. (measure twice)

**Recording**

Bend down if necessary using safe work practices to read the measurement at eye level.

1. Perform HH
2. Record height to the nearest 1mm (0.1cm)
3. Record measurements and any notes on measurement form/Ipad in Redcap

**Cleaning of Equipment**

1. Wipe scales and measurement head plate with neutral detergent wipes and allow to air dry after each use.
2. Packing equipment for transport & carrying
3. Perform HH.
